# Fibre tract segmentation for intraoperative diffusion MRI in neurosurgical patients using tract-specific orientation atlas and tumour deformation modelling

**DOI:** 10.1007/s11548-022-02617-z

**Published:** 2022-04-25

**Authors:** Fiona Young, Kristian Aquilina, Chris A. Clark, Jonathan D. Clayden

**Affiliations:** 1 Institute of Child Health, University College London, Guilford Street, London, United Kingdom; 2 Department of Neurosurgery, Great Ormond Street Hospital for Children, Great Ormond Street, London, United Kingdom

**Keywords:** Intraoperative, Diffusion, MRI, White matter segmentation

## Abstract

**Purpose:**

Intraoperative diffusion MRI could provide a means of visualising brain fibre tracts near a neurosurgical target after preoperative images have been invalidated by brain shift. We propose an atlas-based intraoperative tract segmentation method, as the standard preoperative method, streamline tractography, is unsuitable for intraoperative implementation.

**Methods:**

A tract-specific voxel-wise fibre orientation atlas is constructed from healthy training data. After registration with a target image, a radial tumour deformation model is applied to the orientation atlas to account for displacement caused by lesions. The final tract map is obtained from the inner product of the atlas and target image fibre orientation data derived from intraoperative diffusion MRI.

**Results:**

The simple tumour model takes only seconds to effectively deform the atlas into alignment with the target image. With minimal processing time and operator effort, maps of surgically relevant tracts can be achieved that are visually and qualitatively comparable with results obtained from streamline tractography.

**Conclusion:**

Preliminary results demonstrate feasibility of intraoperative streamline-free tract segmentation in challenging neurosurgical cases. Demonstrated results in a small number of representative sample subjects are realistic despite the simplicity of the tumour deformation model employed. Following this proof of concept, future studies will focus on achieving robustness in a wide range of tumour types and clinical scenarios, as well as quantitative validation of segmentations.

## Introduction

Neurosurgery carries risks to healthy brain structures, including neuron fibre bundles called white matter tracts, injury to which can cause disruption to such important functions as movement, vision and speech. The spatial relationship between neurosurgical targets and adjacent white matter tracts can be determined preoperatively from diffusion magnetic resonance imaging (dMRI). However, the information in preoperative images becomes inaccurate as the spatial relationships change over the course of surgery (brain shift).

Intraoperative dMRI offers a means for imaging fibre tracts after brain shift has invalidated preoperative imaging. However, the unique challenges of intraoperative imaging, which include strict time constraints on image acquisition and post-processing , and on the availability of specialist operators and computing equipment, mean that standard preoperative image processing techniques do not translate easily to the intraoperative environment.

The current clinical standard for reconstructing tracts from dMRI data preoperatively is streamline tractography [1, 2], in which fibre tracking algorithms generate virtual fibres from fibre orientations modelled from dMRI data. However, there remains a notable gap between advanced tractography and tract segmentation methods widely used in dMRI research and those methods that remain commonplace in clinical practice, despite clear evidence of the accuracy and reliability drawbacks characteristic of the latter more outdated tractography techniques [3]. A major factor behind this adoption delay is likely convenience: implementation of streamline tractography can be time-consuming, and obtaining accurate results in the presence of tumours is difficult [4]. Generating reconstructions of specific tracts requires (usually manual) placement of anatomical regions of interest, as well as manual post-processing to remove spurious streamlines. In addition, tractography has poor reproducibility, with results depending on numerous factors [5]. All considered, it is perhaps unsurprising that there is hesitancy within the neurosurgical community to adopt intraoperative tractography [6], even as interest in intraoperative MRI [7, 8] and tractography for surgical planning and navigation [9, 10] grows. Currently intraoperative streamline tractography is mostly limited to the often outdated tools available in commercial navigation software [3]. For example, the iPlan FibreTracking module in Brainlab surgical navigation tools (Brainlab, Feldkirchen, Germany) uses FACT (fibre assignment by continuous tracking), a deterministic, diffusion tensor derived tracking algorithm first proposed in 1998 [11, 12]. That is not to say it is appropriate to simply update the tractography techniques implemented in commercial tools. In general, the ill-posed nature of tractography results in a trade-off between sensitivity and specificity, with those methods common in clinical use generally exhibiting low spatial coverage of tracts (low sensitivity), while state-of-the-art tractography is afflicted by high numbers of spurious streamlines (low specificity) [1], which could unhelpfully obscure intraoperative navigation. Instead, there is a need for alternative tract segmentation methods that do not directly utilise streamline tractography. For example, TractSeg, a deep neural network model for direct tract segmentation, has been proposed for use in neurosurgical patients [13]. TractSeg does not incorporate any explicit handling of lesion mass-effects, leading to partially incomplete segmentations in some cases. We propose an atlas-based method, “tractfinder”, with patient-specific lesion deformation modelling.

One difficulty of using atlas-based segmentation methods in clinical subjects is that of anatomical non-correspondence between subject and template images caused by space-occupying lesions. Deformable registration alone is often insufficient for handling this mismatch [14], and so using tumour growth models to simulate the deformation in the atlas prior to registration is the commonly preferred approach [15, 16]. Numerous previously proposed tumour deformation models aim to achieve highly accurate modelling of tumour growth dynamics and the effects on surrounding tissues, by taking into account elastic tissue properties and microscopic tumour growth modelling. The resulting algorithms are mathematically complex [14], require optimisation of tumour parameters through problem inversion or by other means [16–19] and take anywhere between 1 and 36 hours to run [18, 20–23], even on high-performance computing setups.

Given the time constraints of intraoperative imaging and the practical constraints of the computing capacity which can reasonably be assumed to be available in an operating room, our aim is to achieve an estimate of tract displacement with low computational complexity. The first component of tractfinder, the tract orientation atlas, provides a degree of spatial tolerance that alleviates the need for voxel-perfect registration and deformation, allowing the implementation of a minimal deformation algorithm. We then derive a tract segmentation from the overlap between the deformed atlas and fibre orientation information in the target image.

The novel contributions of this work are explicit handling of large-scale deformations and an automated pipeline that can produce results within 15 minutes. The pipeline can be run fully automatically with no minimal to no user input, depending on the particularities of an individual case (such as lesion mass effect and extent of resection). Tractfinder has been developed specifically for intraoperative imaging, but is equally applicable to any diffusion MRI data.

## Methods

The tractfinder pipeline consists of three main components. The first component, the tract atlas, acts as a prior on a tract’s spatial location and orientation. It incorporates known knowledge about tracts in a way similar to the use of regions of interest in tractography. Next, tumour deformation modelling of the atlas corrects for the displacement of tracts by space-occupying lesions. Minimal adjustment to precomputed deformations can account for intraoperative brain shift. Finally, the deformed tract atlas and target dMRI fibre orientation data are combined to compute a likelihood map for the tract.

Since the focus of this communication is on tumour deformation modelling, the other components will only be briefly summarised for completeness. Throughout this section, wherever there is talk of an orientation distribution, all such distributions are represented in spherical harmonic (SH) basis [24]. In this framework, a distribution is parameterised by its SH coefficients up to a maximum order set to $l_{max}=8$ unless otherwise specified.

### Tract orientation atlas

A separate atlas is created for each tract of interest to capture its typical orientation and location. To date, bilateral atlases have been constructed for the optic radiation and corticospinal tract. First, the tract is reconstructed in each of a series of healthy training dMRI datasets (n=16) [25] using multi-shell multi-tissue constrained spherical deconvolution [26–28] and probabilistic streamline tractography [29, 30] and a consistent ROI-based reconstruction protocol. After manual filtering of biologically implausible streamlines, the reconstructions are transformed to MNI space [31].

Next, tract orientation distribution (TOD) mapping [32] is used to calculate the distribution of streamline orientations within each voxel. The TOD map is normalised to unit integral on the sphere in order to remove streamline density information. Finally, the mean over all training normalised TOD maps is computed.

The resulting tract atlas is an average map of the tract over all training subjects, which contains both a spatial and orientational components. For use in unseen target subjects, the tract atlas is linearly registered to the target image for subsequent calculations.

### Tumour deformation modelling

The tract orientation atlas represents the expected orientation and location of the tract for a typical healthy subject. In order to correct for displacement of white matter tracts due to space occupying lesions, a simple radial tumour expansion model is employed.

**Fig. 1:**
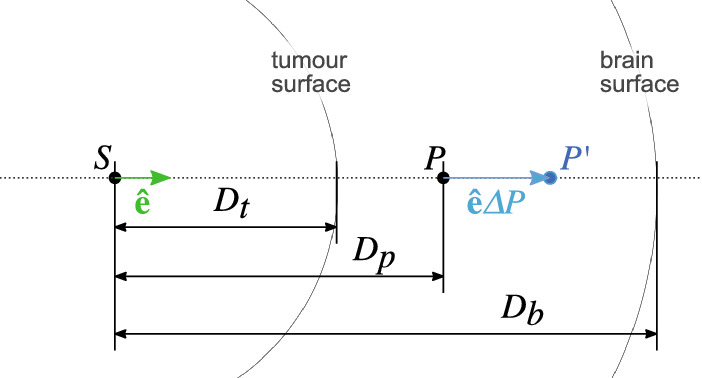
Schematic of variables in radial tumour expansion model

The model has been adapted from the one described by Nowinski and Belov [33]. The model inputs are the segmentations of the tumour and brain volumes. Tumour segmentations were drawn manually for this study, while brain masks are readily computed by available MRI analysis software [28, 30, 34]. We define the direction ${\hat{\mathbf {e}}}$, which is the unit vector along the line connecting a point *P*(*x*, *y*, *z*) to the tumour centre of mass, *S*. Along ${\hat{\mathbf {e}}}$ we also define $D_p$ as the distance $\Vert \overrightarrow{SP}\Vert$, $D_b$ as the distance from *S* to the brain surface and $D_t$ as the distance from *S* to the tumour surface (Fig. 1).

Then, for a point in the original image $P = (x,y,z)$ the transformed location in the deformed image $P' = (x',y',z')$ is

1 $$\begin{aligned} P' = f(P) = P + \hat{\mathbf {e}}kD_ts. \end{aligned}$$

**Fig. 2:**
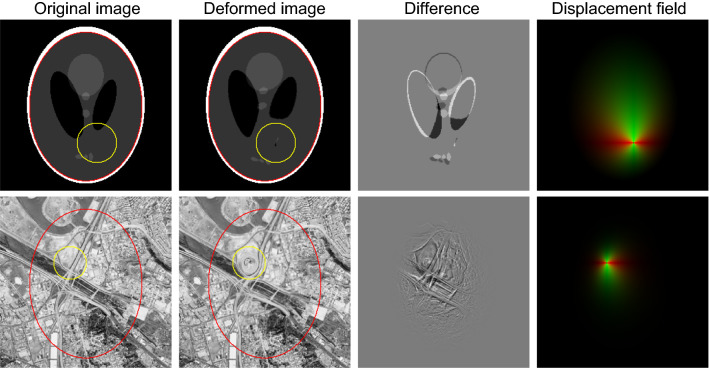
Demonstration of tumour deformation model on standard test images, using different values of $\lambda$. Larger values of $\lambda$ result in more localised deformation fields, while normalisation ensures deformation is always zero at the brain boundary (red ellipse). Yellow circle = simulated tumour boundary. Top: Shepp–Logan phantom, $\lambda = 2$. Middle: San Diego aerial test image, $\lambda = 6$

An exponentially decaying function is used to model the displacement of each voxel (Fig. 2). This choice was made in contrast to the linear relationship used in [33] as it provides a better approximation to typically observed tumour displacement patterns, while remaining an easily computable, closed-form and invertible function. The amount of displacement depends exponentially on the relative distance to the tumour and brain surfaces via the following relationship:

2 $$\begin{aligned} k(P) = (1-c)e^{-\lambda \frac{D_p}{D_b}} +c, \end{aligned}$$

where the normalisation constant $c = \frac{e^{-\lambda }}{e^{-\lambda }-1}$ ensures that $k = 1$ when $D_P = 0$ and $k = 0$ when $D_p = D_b$. The appropriate value for the decay parameter $\lambda$ will depend on the specific lesion being modelled. For example, smaller lesions (20-30mm diameter) typically displace tissue only in their immediate surroundings, with distant tissue remaining virtually unmoved. In such cases, a higher value of $\lambda$ ($\ge 3$), indicating stronger decay of deformation, would be appropriate (Fig. 1).

Equations (1) and (2) describe the deformation field in forward warp convention. To deform an image using reverse warp (“pull-back”) convention, the inverse mapping $P' = f^{-1}(P)$ is needed, which is obtained by solving equation (1) for *P*:

3 $$\begin{aligned} P = P' - \hat{\mathbf {e}}\left( D_t c - \frac{D_b}{\lambda }\mathcal {W}_0\left( \frac{-\lambda D_t (1-c) e^{-\lambda (D_p'-D_tc)/D_b}}{D_b}\right) \right) , \end{aligned}$$

where $\mathcal {W}_0(y)$ is the principal branch of the Lambert $\mathcal {W}$ function, defined as the inverse function of $y(x) = xe^x$ for $x,y \in \mathbb {R}$.

If the lesion is not invading the surrounding tissue but instead fully displacing it (non-infiltrative), then under the simplified assumption that no original, healthy tissue is destroyed, $\lambda$ should be set to a value that ensures that every point *P* within the lesion boundary is displaced to a new position $P'$ that is strictly outside the boundary. In other words,

4 $$\begin{aligned} k(P) = (1-c)e^{-\lambda \frac{D_P}{D_b}} +c \ge 1 - \frac{D_P}{D_t} \end{aligned}$$

must hold for all *P*.

Given that the gradient of *k* is strictly decreasing and $g(D_P) = 1 - \frac{D_P}{D_t}$ is linear, it is sufficient to set

5 $$\begin{aligned} \frac{d}{dP}\bigg |_{D_P=0}k(D_P) = \frac{d}{dP}\bigg |_{D_P=0}g(D_P). \end{aligned}$$

Differentiating both functions at $D_P=0$ and solving for $\lambda$, we have $\lambda _{max} = \frac{D_b}{D_t (1-c)}$. Thus, for strictly non-infiltrating lesions, we set $\lambda \le \lambda _{max}$ to satisfy equation (4), where $\lambda _{max}$ is used as the default value if none is specified. Note that $\lambda _{max}$ varies throughout the brain, as it depends on the relative distances to brain and tumour surfaces for each specific *P*.

**Fig. 3:**
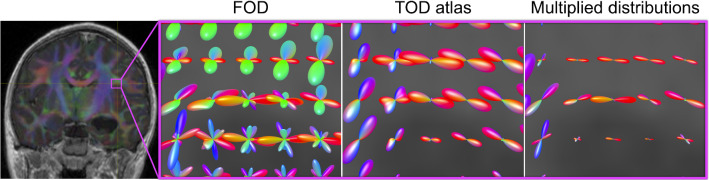
Illustration of atlas and FOD combination, with close-up of a crossing region between the corticospinal tract (CST) and association fibres of a separate tract. The crossing fibres are visible as green FOD lobes, while branching CST fibres are represented by the purple and red lobes. Only directions corresponding to CST fibres are present in the TOD atlas. The multiplication of the two distributions results in suppression of non-CST signal. Integrating the multiplied distributions gives the final scalar map (not shown)

The tumour deformation model is implemented in Python, and full execution takes on average 1 min for a 208 x 256 x 256 voxel image. If lookup tables for $$ D_t$$ and $$D_b$$ are precomputed and saved, then subsequent executions of the model (e.g. with different values for $$\lambda $$ and *s*, as appropriate for a given tumour) take less than 10 seconds, as long as the tumour and brain segmentations remain unchanged.

### Prior and data combination

The final step, after registering and deforming the orientation atlas to approximately match the anatomy of the target image, is to compare the expectation represented in the atlas with the observed dMRI data of the target image. Our objective is to obtain a measure per voxel of how closely the predicted tract orientation distribution overlaps with the observed fibre orientation distribution (FOD), which is modelled from the target dMRI data using multi-shell multi-tissue constrained spherical deconvolution (CSD) [26, 27] (Fig. 3).

This can be achieved by taking the inner product of the two functions, i.e. multiplying them and integrating the product over all spherical angles. The FOD and TOD atlas are both represented by their spherical harmonic (SH) distribution functions as follows:

6 $$\begin{aligned} F(\theta , \phi ) = \sum _{l=0}^{l_{max}} \sum _{m=-l}^l f_{l,m} Y_{l,m}(\theta , \phi ) = \sum _j f_jY_j(\theta , \phi ), \end{aligned}$$

where $Y_{l,m}$ is the modified SH basis described in [24]. The spherical integral of the product of two spherical harmonic basis functions is $\int _0^{\pi } \int _0^{2\pi } Y_{l_1,m_1}(\theta , \phi ) Y_{l_2,m_2}(\theta , \phi ) sin(\theta ) d\theta d\phi = \delta _{m_1, m_2} \delta _{l_1, l_2}$. Therefore, for two functions $F(\theta , \phi )$ and $G(\theta , \phi )$ the integral of their product can be expressed as:

7 $$\begin{aligned} \begin{aligned}&\int _0^{\pi } \int _0^{2\pi } F(\theta , \phi ) G(\theta , \phi ) sin(\theta ) d\theta d\phi \\&\qquad = \int _0^{\pi } \int _0^{2\pi } \left( \sum _j f_jY_j(\theta , \phi )\right) \left( \sum _k g_kY_k(\theta , \phi )\right) \\&\quad \qquad \qquad \times sin(\theta ) d\theta d\phi \\&\qquad = \sum _{j,k} f_j g_k \delta _{jk}. \end{aligned} \end{aligned}$$

Thus, for two distributions represented by a vector containing their spherical harmonic coefficients, the integrated product can be obtained by taking the inner product of the two coefficient vectors.

### Application to intraoperative dMRI

The methodology described above was initially developed and tested in preoperative tumour images. However, the target application is in intraoperative imaging. The main difference therein is the need to account for brain shift, which is unpredictable: differing effects stem from drainage of fluid, pressure changes, tumour debulking and gravitational sag. Nevertheless, we aim to achieve intraoperative tract segmentation while avoiding the need to perform additional tumour and/or resection cavity segmentation intraoperatively.

As the atlas is designed to be spatially inclusive, with the inner product acting to correct small spatial inaccuracies, it is possible in some cases where brain shift is minimal to reuse the preoperative tumour deformation field. In cases of significant tumour debulking, the deformation field can be recomputed from the preoperative tumour segmentation by adjusting the value of *s* to simulate a reduction in tumour volume.

**Fig. 4:**
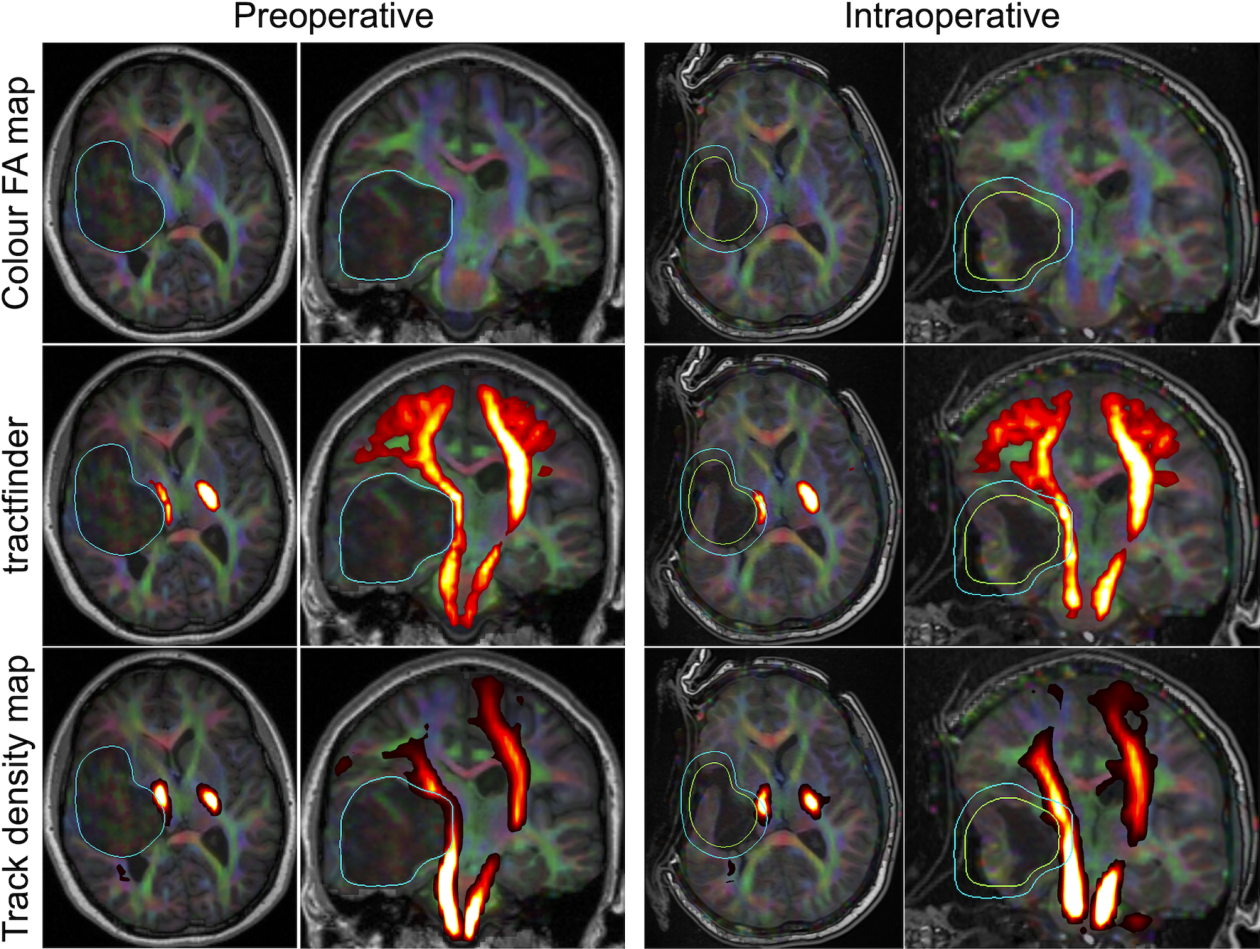
Example results in intraoperative image using scaled preoperative tumour segmentation. Blue outline: Tumour segmentation. Green outline: effective tumour boundary with $s=0.8$ used for intraoperative segmentation

This scenario is demonstrated in Fig. 4, showing the resection of a large temporal epidermoid cyst. There is significant reduction in cyst volume and the adjacent corticospinal tract has shifted accordingly; however, by reusing the preoperative lesion segmentation and setting $s=0.8$, the resulting deformation field is able to capture the rough location of the shifted tract. By only adjusting the value of *s* and reusing preoperatively computed values of $D_t$ and $D_b$, we can avoid time and resource-intensive intraoperative lesion segmentation, brain shift modelling or nonlinear registration.

## Results and discussion

**Fig. 5:**
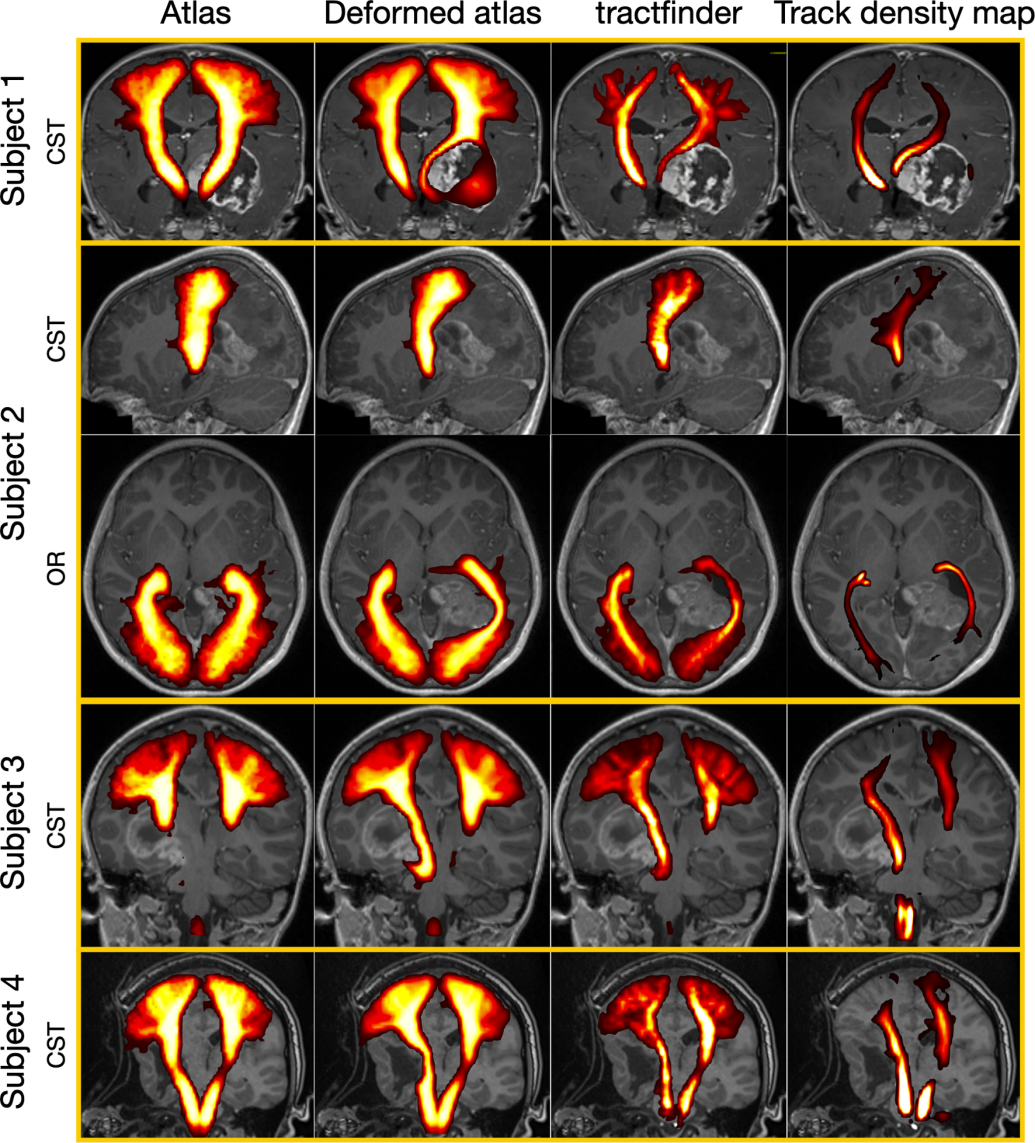
Sample results in 4 different clinical subjects (1: unknown, 2: 11y F, 3: 5y M, 4: 30y F). First column: linearly registered tract atlas (spatial component only). Second column: atlas after tumour deformation. Third column: Final tract map. Fourth column: Track density image from streamline tractography, where intensity corresponds to streamline count per $(2.5 mm)^3$ voxel (thresholded at 10 streamlines). In each subject *j*, the value of $\lambda$ varies spatially and was set automatically to $min\{\lambda _j, \lambda _{max}\}$ according to equation (4), with $\lambda _j=8$ everywhere except for subject 2, optic radiation, where $\lambda _j=2$. In subjects 1-3 $s=1$, in subject 4 $s=0.8$ (see also Figure 4). Key: CST = corticospinal tract, OR = optic radiation

Due to the lack of ground truth information for white matter tract segmentation in *in vivo* dMRI images, especially in neurosurgical cases, a quantitative validation of this method is not currently possible. However, comparison with the current clinical standard, streamline tractography, illustrates the effectiveness of tractfinder. Targeted probabilistic streamline tractography reconstructions were produced by an experienced operator utilising ROI placement strategies routinely utilised in clinical practice at our institution. The clinical reliability and biological accuracy of tractography is difficult to determine *in vivo*. While some consider the “gold standard” to be validation against intraoperative direct electrical stimulation and post-surgical outcomes [3], which is unavailable for the presented data, even this can provide only incomplete information on spatial accuracy. Therefore, a quantitative comparison between the proposed methodology and streamline tractography is not available at present, with comprehensive validation remaining the subject of future investigations. Figure 5 shows results for four different example subjects (three paediatric and one adult) with space occupying tumours. These initial results serve to demonstrate the feasibility of the proposed method (demonstrated in the corticospinal tract in all four subjects and additionally the optic radiation in subject 2) in a range of complex clinical cases.

The tumour deformation model successfully captures large-scale tract displacements in seconds, where much longer timescales (several minutes to hours) are typical for more complex tumour growth modelling algorithms and nonlinear registration. The short computational time further makes it trivial to recompute the deformation with small adjustments if necessary. The model presents a simplified prediction of tumour deformation: No distinctions are made between the highly deformable ventricles and stiffer brain tissues, and the tumour is “grown” isotropically from a single point outward with no regard for the surrounding topology (except for the brain boundary) or peri-tumoural tissue effects. Nevertheless, the objective of the deformation step, which is to bring the tract orientation atlas into approximate alignment with the actual target tract, is achieved despite these simplifications. Improvement is needed in cases involving infiltrative tumours, where tracts are not entirely displaced and tumour cells mix with surrounding functional structures, as the current model only supports single tumours with defined boundaries. Modelling this scenario will require a modified deformation model, using a different expression for *k*(*P*).

The inner product between the orientation atlas and target FOD image provides an intuitive map of tract location and is computationally straightforward (Figs. 3 and 5). Successful results have been obtained in clinical quality, single-shelled diffusion MRI datasets (see Appendix 1). However, there remains the need to more thoroughly explore the effects of different acquisition protocols, including fewer diffusion encoding directions and lower *b*-values, on segmentation quality. So far, there has been limited validation of applying tractfinder to intraoperative cases, and this will be the subject of future research. One example of such a case is shown in Fig. 4. Using a lesion shrinkage factor of $s=0.8$ is successful at creating a deformation field that corresponds with the intraoperative anatomy, and the resulting map of the CST captures the tract’s course at the edge of the lesion and resection cavity.

If implemented clinically, intraoperative processing steps would be limited to minimal preprocessing including de-noising [30, 35, 36] and bias field correction [30, 37], registration to preoperative data [38], followed by FOD modelling [27, 39], adjustments to tumour deformation modelling if necessary and inner product computation. Other preprocessing steps which are routine in preoperative and research imaging contexts, such as correction for eddy current and geometric distortion artefacts, have been omitted due to long processing times making them impractical for intraoperative use. Future research should investigate the implications of omitting these corrections and possible more lightweight implementations. Total processing time for the above steps should not exceed 15 minutes, and could be completed in parallel with the non-diffusion iMRI acquisition protocol (if the site-specific setup allows parallel acquisition and data processing), which can take up to 50 minutes. Operator input and time is currently required preoperatively for manual tumour segmentation, although this step could feasibly be automated given the extensive interest and research into automatic brain tumour segmentation [40, 41]. Additionally, all intraoperative processing steps can be completed automatically using default values. The process is no longer fully automatic if manual adjustments to $\lambda$ and *s* are necessary; however, this nevertheless amounts to far less operator input than advanced streamline tractography as described in the introduction. A final critical practical aspect of intraoperative implementation will be the integration of segmentation results with neuronavigation tools for display during surgery, including the appropriate data conversions. Tools to enable such interfacing have been developed by others [42, 43] and could likely form part of the full clinical tractfinder implementation.

In conclusion, a white matter mapping method is presented that is shown to produce plausible tract reconstructions in cases with space occupying lesions, using an atlas in conjunction with tumour deformation modelling. Producing results requiring minimal user input and on intraoperatively feasible timescales, the method thus has the potential to bring effective white matter mapping into the intraoperative domain.

## Imaging data parameters

The results displayed in Fig. 5 are derived from diffusion MRI datasets with the following parameters:

**Table:**

Subject	Shell *b*-value(s)	Number of directions
1	1000, 2200	60, 60
2	1000	60
3	1000	60
4	1000	36
