# Correction to: Fibre tract segmentation for intraoperative diffusion MRI in neurosurgical patients using tract-specific orientation atlas and tumour deformation modelling

**DOI:** 10.1007/s11548-022-02667-3

**Published:** 2022-05-26

**Authors:** Fiona Young, Kristian Aquilina, Chris A. Clark, Jonathan D. Clayden

**Affiliations:** 1 Institute of Child Health, University College London, Guilford Street, London, United Kingdom; 2 Department of Neurosurgery, Great Ormond Street Hospital for Children, Great Ormond Street, London, United Kingdom

## Correction to: International Journal of Computer Assisted Radiology and Surgery 10.1007/s11548-022-02617-z

The original version of this article unfortunately contained a mistake. Affiliation details for Author Chris A. Clark were incorrectly given as

Department of Neurosurgery, Great Ormond Street Hospital for Children, Great Ormond Street, London, United Kingdom

but should have been

Institute of Child Health, University College London, Guilford Street, London, United Kingdom

The original article has been corrected.

